# Characterization of *Burkholderia rhizoxinica* and *B. endofungorum* Isolated from Clinical Specimens

**DOI:** 10.1371/journal.pone.0015731

**Published:** 2011-01-18

**Authors:** Jay E. Gee, Mindy B. Glass, Gerald Lackner, Leta O. Helsel, Maryam Daneshvar, Dannie G. Hollis, Jean Jordan, Roger Morey, Arnold Steigerwalt, Christian Hertweck

**Affiliations:** 1 Bacterial Special Pathogens Branch, Division of High-Consequence Pathogens and Pathology, National Center for Emerging and Zoonotic Infectious Diseases, Centers for Disease Control and Prevention, Atlanta, Georgia, United States of America; 2 Leibniz Institute for Natural Products Research and Infection Biology, Jena, Germany; 3 Friedrich Schiller University, Jena, Germany; Institut de Pharmacologie et de Biologie Structurale, France

## Abstract

Eight isolates submitted to CDC from 1989 to 2006 from clinical specimens were initially identified as members of the genus *Burkholderia* based on preliminary cellular fatty acid analysis and/or 16S rRNA gene sequencing. With the recent descriptions of the new species *B. rhizoxinica* and *B. endofungorum*, which are considered endosymbiotic bacteria in *Rhizopus microsporus* fungi, we now identify seven of these clinical isolates as *B. rhizoxinica* and one as *B. endofungorum* based on biochemical testing, 16s rRNA, and DNA-DNA hybridization results. We also further characterize these isolates by assessing toxin production and/or by multiple locus sequence typing.

## Introduction

Recently, *Burkholderia* spp. have been described that are not only endosymbionts in *Rhizopus microsporus*, a saprotrophic fungi, but are also responsible for the production of the toxins rhizoxin and rhizonin which had been previously believed to have been produced solely by the fungi [1], [2], [3]. Rhizoxin is a important virulence factor for infection of plants by *Rhizopus* and has antimitotic activity [4], [5], [6]. Rhizonin is a cyclopeptide exhibiting fatal hepatotoxic effects [3], [5], [7], [8]. *Burkholderia rhizoxinica* produces rhizoxin and is now associated with the ability of *Rhizopus* to cause rice seedling blight. *Burkholderia endofungorum* was recently shown to produce rhizonin. Both bacteria appear to reside in the cytosol of the fungal cell [6], [9].

Clinical isolates H2199 (Ohio - 2002), H3620 (New Mexico - 2005), H500 (California - 1997), G8810 (North Carolina - 1993), G7344 (Oregon - 1992), H2592 (District of Columbia - 2003), H3977 (South Dakota - 2006), and G4101 (New York - 1989) were submitted to the CDC from 1989 to 2006 by various public health agencies in the U.S. and placed in our archive. All but one were isolated from blood specimens with the exception being H2592 which was from a wound. These isolates were obtained from five adult males and three adult females. Later enquiry on the clinical background of these isolates did not yield any further information. Biochemical characterization was not able to provide identification; however, cellular fatty acid analysis of H500, G7344, G8810 and G4101 indicated profiles similar to that for *Burkholderia pseudomallei* and/or the *Burkholderia cepacia* complex. Analysis of the 16S rRNA gene sequences also indicated that these isolates were members of the genus *Burkholderia*, but did not yield a specific identity.

Based on the new descriptions of *B. rhizoxinica* and *B. endofungorum*, we re-examined the isolates in our archive using standard biochemical testing, 16S rRNA gene sequencing and DNA-DNA hybridization. We now identify these clinically derived strains as either *B. rhizoxinica* or *B. endofungorum*. We also further characterize them by cellular fatty acid analysis (CFA) and/or and multiple locus sequence typing (MLST).

## Materials and Methods

Biochemical testing was performed on all strains and cellular fatty acid (CFA) analysis was performed on strains H500, G7344, G8810 and G4101 as described by Weyant *et. al.* [10].

We performed DNA-DNA hybridization as previously described [11]. In brief: Cells were harvested and lysed, and the chromosomal DNA was isolated and purified. DNA from the type strains of *B. rhizonica* (HKI 454^T^) and *B. endofungorum* (HKI 456^T^) were labeled with [^32^P]dCTP using a commercial nick translation kit (Invitrogen Life Technologies, Carlsbad, CA) and tested for reassociation to unlabeled DNA from the same strains (homologous reaction). Reassociation of *B. rhizonica* DNA was tested with DNA from H3977 and H2199 and that of *B. endofungorum* was tested with DNA from G4101. A reciprocal reaction using labeled G4101 DNA was also performed. Relative binding ratios and percent divergence were calculated as described previously [11].

DNA sequencing was performed using methods and primers as previously described. In brief: whole cell suspensions of bacteria were used for PCR. Bacteria were grown by plating one loop (1 µl) of stock cell suspension (heavy suspension of *Burkholderia* spp. in defibrinated rabbit blood, stored at −70°C until use) on trypticase soy agar with 5% defibrinated sheep blood (SBA) (BBL Microbiology systems, Cockeysville, MD) and incubating aerobically 1–2 days at 37°C. A single colony was suspended in 200 µl of 10 mM Tris, pH 8.0 in a 1.5 ml Millipore 0.22 µm filter unit (Millipore, Bedford, MA), heated at 95°C for 30 min, and centrifuged at 6000× g for 5 min. Each final PCR reaction (100 µl) contained 5 U of Expand DNA polymerase (Boehringer Mannheim, Mannheim, Germany), 2 µl of DNA solution in H_2_O, 10 mM Tris-HCl (pH 8.0), 50 mM KCl, 1.5 mM MgCl_2_, 200 µM (each) dATP, dCTP, dGTP, and dTTP, and 0.4 µM of each primer. Reactions were first incubated for 5 min at 95°C. Then, 35 cycles were performed as follows: 15 sec at 94°C, 15 sec at 60°C, and 1 min and 30 sec at 72°C. Reactions were then incubated at 72°C for an additional 5 min. PCR products were purified with Qiaquick PCR purification kit (Qiagen, Valencia, CA). Sequencing was performed using an Applied Biosystems (ABI) BigDye terminator cycle sequencing ver 3.1 kit as per the manufacturer's instructions, except 0.25 µl of BigDye were used instead of 8 µl (Applied BioSystems, Foster City, CA). Sequencing products were purified by using Centri-Sep spin columns (Princeton Separations, Adelphia, NJ), and were resolved using an Applied Biosystems model 3130xl automated DNA sequencing system (Applied Biosystems) [9], [12], [13], [14]. Analysis was performed using the Accelrys GCG package ver 10.3 (Accelrys, San Diego, CA) and MEGA 3 as previously described [14], [15].

The 16S rRNA gene sequences obtained for strains G7344, G8810, H500, H2199, H2592, H3620, H3977 and G4101 were deposited in GenBank (Supporting Information S1). Analysis of 16S rRNA sequences were performed as previously described [13]. A reconstruction of the phylogenetic relationship of the bacterial isolates by multi-locus sequence typing (MLST) based on six gene loci (*gltB*, *gmhD*, *lepA*, *lipA*, *ndh*, and *rhiE*) was performed as previously described [9] and sequences were deposited in GenBank (Supporting Information S1).

The obtained dataset consisted of 3160 nucleotides including 710 variable sites, 263 of them being parsimony informative. Phylogenetic analyses were carried out in a Bayesian and distance matrix framework. For Bayesian analyses we employed the GTR+I+G model of nucleotide evolution. Bayesian tree sampling was performed using the MrBAYES 3.1.1 program [16], [17]. Two analyses including 500,000 generations each were run in parallel. Four chains were run simultaneously. Trees were sampled every 100th generation. The first 3,000 generations were deleted as the “burn in” of the chain. The remaining trees were summarized as 50% majority rule consensus trees. Phylogenetic trees were visualized using the program Treeview [18]. Neighbor joining trees were constructed using MEGA version 3.1 [15]. Branch support for NJ trees was obtained by performing 10000 bootstrap replicates. The corresponding *Burkholderia pseudomallei* ST1 (BPS) sequence was used as an outgroup to root the tree. For the construction of phylogenetic networks with the neighbor-net method we used the program SplitsTree version 4.8 [19].

For secondary metabolite analysis the bacterial strains were cultured on nutrient agar plates at 30°C. After 3 days a single colony was used to inoculate 1 ml TSB. After 2 days cultivation at 30°C and 120 rpm, 1 ml fresh TSB was added. After another 2 days cultivation, the grown culture was transferred to 20 ml TSB and again incubated at 30°C (120 rpm) for 48 h. An aliquot of 5 ml was used to inoculate 100 ml of production medium (1% corn starch, 0.5% glycerol, 1% gluten meal, 1% dried yeast, 1% corn steep liquor, and 1% CaCO3, pH = 6.5). Cultivation took place at 30°C, 120 rpm, for 4 days.

Extraction and HPLC analysis was performed as described previously [20].

## Results

These eight clinical isolates shared common phenotypic characteristics. They were nonmotile, gram-negative, coccobacilli. They produced oxidase but did not produce catalase, indole, urease, did not reduce nitrite, did not hydrolyze gelatin or esculin, and did not utilize citrate. Nitrate was reduced by only one isolate H3977. No acid production in the slant or butt of triple-sugar iron agar was noted. There was no growth on MacConkey agar, Salmonella Shigella agar or on cetrimide agar. No acid production was observered in King's oxidation-fermentation base from D-glucose, D-xylose, mannitol, lactose, sucrose, and maltose. Variable reactions were produced with litmus milk. We were unable to demonstrate catalase production and motility with neither of these clinical strains or with the type strains of *B. rhizoxinica* (HKI 454^T^) and *B. endofungorum* (HKI 456^T^) in our laboratory even though these characteristics were reported by Partida-Martinez and coworkers [6]. Partida-Martinez and coworkers noted that growth of these bacteria was poor in pure culture and did not allow for consistent biochemical characteristics [6].

**Figure 1:**
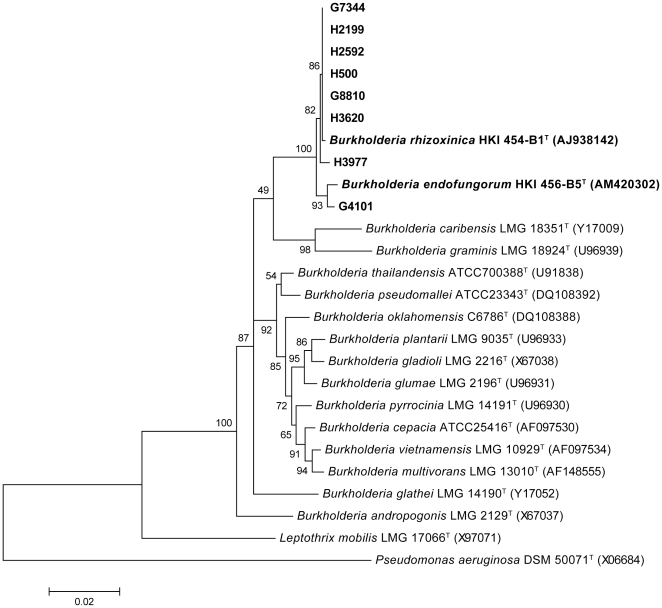
MEGA 3 analysis of 16S rRNA gene sequences using neighbor-joining and Kimura 2-parameter settings with 1000 step bootstrap. Phylogenetic tree of *Burkholderia rhizoxinica* and *B. endofungorum* based on comparisons with 16S rDNA sequences of related bacteria. *Pseudomonas aeruginosa* DSM 50071 (GenBank accession no. X06684) was used as an outgroup for this analysis. Bar, 2% sequence dissimilarity.

Analysis of the 16S rRNA gene sequences indicates that H2199, H3620, H500, G8810, G7344, H2592, H3977 have >99.4% identity to the 16S rRNA gene sequence for the *B. rhizoxinica* type strain (HKI 454^T^) and G4101 has an identity of 99.4% to the sequence for the *B. endofungorum* type strain (HKI 456^T^) (Figure 1).

Four strains were selected for cellular fatty acid analysis (CFA). H500, G7344, G8810 and G4101 shared a unique profile which is easily recognized by the presence of two cyclopropane acids (17:0cyc, 19:0cyc11-12), 16:0 and 18:1w7c as major acids (8–27%), and smaller amounts (1–5%) of six hydroxy acids (3-OH-14:0, 2-OH-16:1, 2-OH-16:0, 3-OH-16:0, 2-OH-18:1, 2-OH-19:0cyc). The CFA composition of H500, G7344, G8810 and G4101 is consistent with that of the type strains of *B. rhizoxinica* and *B. endofungorum* [6], and is most similar to that of the CFA group containing *B. cepacia*, *B*. *gladioli*, *B*. *mallei* and *B*. *pseudomallei* [10].

The results of DNA relatedness studies are given in Table 1. Isolates H3977 and H2199 exhibited greater than 78% relatedness (RBR) under both the optimum and stringent reassociation criteria and had divergence (D) of less than 5 to the labeled DNA from the *B. rhizoxinica* type strain. Isolate G4101 exhibited 76% relatedness under optimum criteria, but 69% under the more stringent conditions when matched with labeled DNA from the *B. endofungorum* type strain, however in the reciprocal reaction using labeled G4101 the relatedness is over 70% for both the optimum and stringent reassociation criteria.

**Table 1:** DNA/DNA hybridization.

Labeled* B. rhizoxinica* HKI 454^T^ DNA			
	RBR @ 65°C	D	RBR @ 80°C
*B. rhizoxinica* HKI 454^T^	100	0.0	100
H3977	83	0.0	84
H2199	85	2.5	79

Labeled *B. endofungorum* HKI 456^T^ DNA			
	RBR @ 65°C	D	RBR @ 80°C
*B. endofungorum* HKI 456^T^	100	0.0	100
G4101	76	3.5	69

Labeled G4101 DNA			
	RBR @ 65°C	D	RBR @ 80°C
G4101	100	0.0	100
*B. endofungorum* HKI 456^T^	82	0.1	72

RBR, relative binding ratio; D, percent divergence.

**Figure 2:**
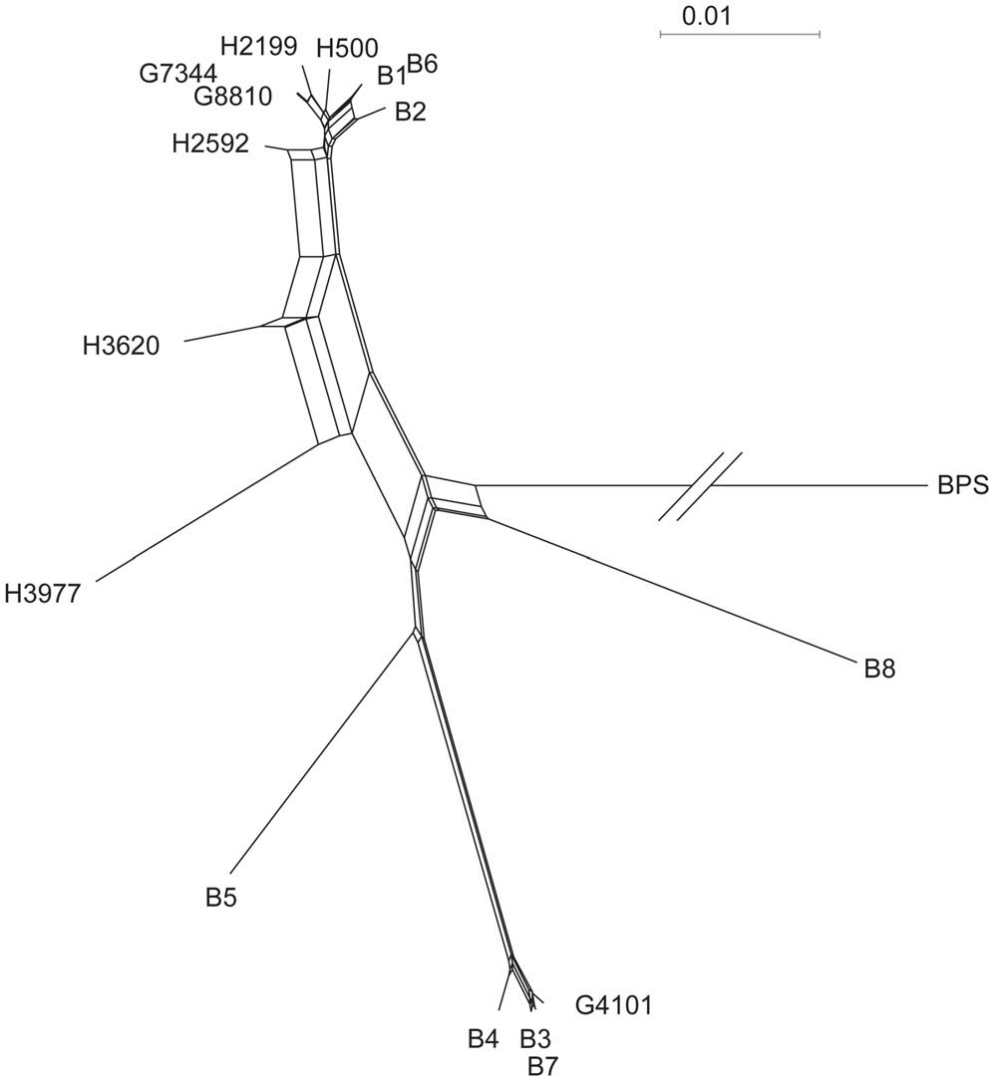
Phylogenetic network of *Burkholderia* spp from. clinical isolates together with fungal symbionts (B1–B8) based on MLST data. B1: *Burkholderia rhizoxinica* type strain. B5: *B. endofungorum* type strain. BPS: *B. pseudomallei* (outgroup).

The results of the MLST indicate that strains G8810, H2190, H500, G7344, H2592, and H3620 are members of the *B. rhizoxinica* clade called the “Pacific group” (97.7% to 99.4% identity with *B. rhizoxinica* type strain), whereas isolate H3977 is more remotely related to this group (95.9% identity) (Figure 2). Strain H3977 is most likely not a member of the known subclades and appears to be the only known member of a new group within the complex. Alleles for isolate G4101 cluster closely with *Burkholderia* spp. from the “Eurasian branch” of endofungal symbionts (99.3% to 99.8% identity) and still has 95.7% identity with the *B. endofungorum* type strain from Mozambique.

**Figure 3:**
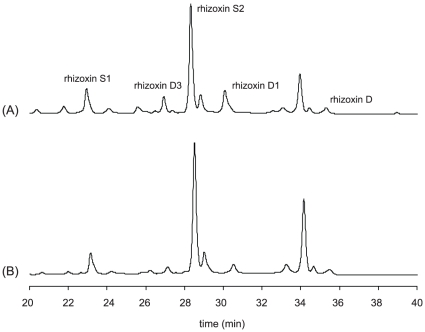
HPLC profiles of culture extracts of strains H2199 (A) and G7344 (B) showing the production of various antimitotic rhizoxin derivatives.

To verify the production of toxins by the clinical isolates the metabolic profiles of two bacterial strains were investigated. Strain H2199 and G7344 proved to be culturable under the conditions optimized for rhizoxin formation whereas we were unable to grow G4101 for the toxin study. HPLC and MS analyses clearly showed that H2199 and G7344 produce high amounts of cytotoxic rhizoxin analogues (Figure 3).

## Discussion

Recently, the toxin rhizonin was shown to be produced by endosymbiont *B. endofungorum* and not by the host *Rhizopus* [3]. It is unknown if the clinical isolate (G4101) of *B. endofungorum* produces rhizonin, but the production of this toxin could be clinically significant since rhizonin is a known hepatotoxin. Further studies will determine if this strain produces rhizonin. However, in all hitherto examined cases there has been no evidence for the involvement of *Burkholderia* symbionts for the development of zygomycoses [21], [22].

The presence of an acyl transferase AT gene (*rhiE*) from the rhizoxin biosynthesis gene cluster suggests that the clinical isolates of *B. rhizoxinica* might have the capacity to produce cytotoxic polyketides [23]. By metabolic profiling of the bacterial cultures, we unequivocally showed that strains H2199 and G7344 produce significant amounts of rhizoxin derivatives. The production of rhizoxin could influence the course of human infection because the toxin has anti-mitotic activity in mammalian cells and has potential as an antitumor drug. Previous work has demonstrated that derivatives of rhizoxin vary in anti-mitotic activity [20]. Further studies will indicate whether these isolates produce the toxin or derivatives of the toxin.

We report for the first time that strains of *B. rhizoxinica* and *B. endofungorum* have been associated with human clinical specimens. Various *Burkholderia* spp. are known pathogens with *B. pseudomallei* causing melioidosis and *B. mallei* causing glanders [13], [24]. There are also cases of opportunistic infection by less pathogenic members of the *Burkholderaea* such as in the case of *B. cepacia* infections, especially among cystic fibrosis patients, and infection by *B. thailandensis* [25], [26].

Since members of the *B. rhizoxinica* complex are known to form tight associations with their host, fungal involvement is possible, however there was no known detection of fungal infections in these cases. Unfortunately the clinical records for these isolates is incomplete and further enquiries did not prove productive. The presence of the bacteria by contamination of the specimens cannot be excluded. Seven of the isolates were derived from blood and one from wound tissue. Since *Rhizopus* are ubiquitous and some are opportunistic human pathogens, it is possible that the patients were colonized by the fungi and that culturing was attempted only to detect bacterial growth, thus missing the presence of *Rhizopus* [27]. When *B. rhizoxinica* or *B. endofungorum* are detected in clinical specimens, clinicians may wish to check for the presence of fungal involvement.

## Supporting Information

Supporting Information S1 **List of genes sequenced with GenBank accession numbers.**
(DOC)
