# Supplementary material for: Characterization of Burkholderia rhizoxinica and B. endofungorum Isolated from Clinical Specimens
Source: PLoS One. 2011 Jan 18;6(1):e15731. doi: 10.1371/journal.pone.0015731 (PMC3022634; doi:10.1371/journal.pone.0015731)
Supplement: Supporting Information S1 — List of genes sequenced with GenBank accession numbers. (DOC) [file pone.0015731.s001.doc]

Supporting Information S1

| Strain | Gene | GenBank Accession Number |
| --- | --- | --- |
| G7344 | 16S | HQ005405 |
| G8810 | 16S | HQ005406 |
| H2199 | 16S | HQ005408 |
| H2592 | 16S | HQ005409 |
| H3620 | 16S | HQ005410 |
| H3977 | 16S | HQ005411 |
| H500 | 16S | HQ005407 |
| G4101 | 16S | HQ005412 |
| G7344 | gltB | HQ130362 |
| G8810 | gltB | HQ130363 |
| H2199 | gltB | HQ130364 |
| H2592 | gltB | HQ130365 |
| H3620 | gltB | HQ130366 |
| H3977 | gltB | HQ130367 |
| H500 | gltB | HQ130368 |
| G4101 | gltB | HQ130405 |
| G7344 | gmhD | HQ130369 |
| G8810 | gmhD | HQ130370 |
| H2199 | gmhD | HQ130371 |
| H2592 | gmhD | HQ130372 |
| H3620 | gmhD | HQ130373 |
| H3977 | gmhD | HQ130374 |
| H500 | gmhD | HQ130375 |
| G4101 | gmhD | HQ130406 |
| G7344 | lepA | HQ130376 |
| G8810 | lepA | HQ130377 |
| H2199 | lepA | HQ130378 |
| H2592 | lepA | HQ130379 |
| H3620 | lepA | HQ130380 |
| H3977 | lepA | HQ130381 |
| H500 | lepA | HQ130382 |
| G4101 | lepA | HQ130407 |
| G7344 | lipA | HQ130383 |
| G8810 | lipA | HQ130384 |
| H2199 | lipA | HQ130385 |
| H2592 | lipA | HQ130386 |
| H3620 | lipA | HQ130387 |
| H3977 | lipA | HQ130388 |
| H500 | lipA | HQ130389 |
| G4101 | lipA | HQ130408 |
| G7344 | ndh | HQ130390 |
| G8810 | ndh | HQ130391 |
| H2199 | ndh | HQ130392 |
| H2592 | ndh | HQ130393 |
| H3620 | ndh | HQ130394 |
| H3977 | ndh | HQ130395 |
| H500 | ndh | HQ130396 |
| G4101 | ndh | HQ130409 |
| G7344 | rhiE | HQ130397 |
| G8810 | rhiE | HQ130398 |
| H2199 | rhiE | HQ130399 |
| H2592 | rhiE | HQ130400 |
| H3620 | rhiE | HQ130401 |
| H3977 | rhiE | HQ130402 |
| H500 | rhiE | HQ130403 |
| G4101 | rhiE | HQ130410 |
